# Microfluidic‐Architected Nanoarrays/Porous Core–Shell Fibers toward Robust Micro‐Energy‐Storage

**DOI:** 10.1002/advs.201901931

**Published:** 2019-11-25

**Authors:** Jinku Meng, Guan Wu, Xingjiang Wu, Hengyang Cheng, Zhi Xu, Su Chen

**Affiliations:** ^1^ State Key Laboratory of Materials‐Oriented Chemical Engineering College of Chemical Engineering Jiangsu Key Laboratory of Fine Chemicals and Functional Polymer Materials Nanjing Tech University Nanjing 210009 P. R. China; ^2^ State Key Laboratory of Chemical Engineering East China University of Science and Technology 130 Meilong Road Shanghai 200237 P. R. China

**Keywords:** fibers, microfluidics, micro‐supercapacitors, nickel oxide arrays, porous graphene

## Abstract

Methods enabling the controllable fabrication of orderly structural and active nanomaterials, along with high‐speed ionic pathways for charge migration and storage are highly fundamental in fiber‐shaped micro‐supercapacitors (MSCs). However, due to fiber‐electrodes with compact internal microstructure and less porosity, MSCs usually display a low energy density. Here, an innovative microfluidic strategy is proposed to design ordered porous and anisotropic core–shell fibers based on nickel oxide arrays/graphene nanomaterials. Owing to the homogeneous microchannels reaction, the graphene core maintains a uniformly anisotropic porous structure, and the nickel oxide shell keeps steadily vertically aligned nanosheets. The MSC presents an ultrahigh energy density (120.3 µWh cm^−2^) and large specific capacitance (605.9 mF cm^−2^). This higher performance originates from the microfluidic‐architected core–shell fiber with abundant ionic channels (plentiful micro‐/mesopores), large specific‐surface‐area (425.6 m^2^ g^−1^), higher electrical conductivity (176.6 S cm^−1^), and sufficient redox activity, facilitating ions with quicker diffusion and greater accumulation. Considering those outstanding properties, a wearable self‐powered system, converting and storing solar energy into electric energy, is designed to light up displays. This microfluidic strategy offers an effective way to design new structural materials, which will advance the development of next‐generation wearable/smart industries.

## Graphical abstract

Microfluidic‐architected ordered porous core–shell fibers of nickel oxide arrays/graphene nanomaterials are fabricated toward robust micro‐energy‐storage. Owing to the homogeneous diffusion, assembly, and reaction in microchannels, the core–shell fibers maintain abundant ionic channels (plentiful micro‐/mesopores), large specific surface area, anisotropic structure, high electrical conductivity, and sufficient redox activity, which endow micro‐supercapacitors with ultrahigh energy density, large specific capacitance, and self‐powered application.

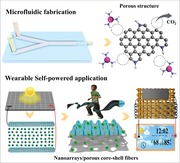

## 1 Introduction

The recent fast progress of advanced energy technologies and wearable industries^1^, ^2^, ^3^ urgently highlights the needs for developing flexible miniaturized energy‐storage devices (MESDs) to power smart electronic products. Specifically, those MESDs can be directly integrated with products to deliver deformable energy supply^4^ in long‐time durability. Among various MESDs, flexible micro‐supercapacitors (MSCs),^5^, ^6^ such as in‐plane on‐chip MSCs and fiber‐shaped MSCs (FMSCs), have triggered a boom because of their tiny volume, lightweight, flexibility, high‐power density, and long cycling stability.^7^, ^8^, ^9^, ^10^ However, one core challenge of flexible MSCs is to largely promote the energy density for delivery or harvesting, or even exceeding the values of microbatteries. Additionally, the ability to develop new functions of MSCs, including weavability, self‐powered feature, and fashion design is still of high demand.

For SCs, the energy storage is mainly ascribed to the formation of electrical double layers (EDLs) and Faradaic redox reaction on electrode–electrolyte interfaces.^11^ In other words, the electrodes materials with ordered porous network, large specific surface area (SSA), electrochemical activity and electrical conductivity are highly important for achieving large electrochemical performance. However, those typical electrode features are primarily predominated by preparation methods because they can manipulate the ordered structure and high electrochemical activity for faster electron conduction and ion diffusion.^4^, ^12^ To this end, many fabrication methods and striking efforts have been realized. For example, on‐chip micro‐energy‐device constructions,^6^, ^13^ including inkjet‐printing,^14^ screen‐printing,^15^ 3D printing,^16^ and spray‐coating^17^ showed high energy density (0.32 µWh cm^−2^)^1^ because of shortening ion transport and diffusion distance. Several spinning approaches, such as wet‐spinning,^8^ dry‐spinning,^18^ and electrospinning^9^ are promising candidates for fabricating fiber‐based electrodes of SCs. They give flexible SCs with not only a large volumetric specific capacitance (177 mF cm^−2^) and energy density (3.84 µWh cm^−2^)^8^ but also excellent bending durability and outstanding weavability. However, the energy density level is still relatively too low to satisfy the practical applications because of the low structural controllability, poor activity, and weaken stability of electrodes.

Currently, microfluidic method has become one of the best method to control the porous structure, morphology, and composition.^19^ Particularly, this method that enables chemical reaction of nanomaterials to be carried out at microscale, allows the homogeneous diffusion and self‐assembly to control ordered structure and activity of fiber‐electrodes.^3^, ^20^ For example, microreactor‐oriented hollow graphene‐based hybrid fibers^21^ with large interfacial surface for electrolyte ions adsorption endowed the MSC a large areal capacitance of 304.5 mF cm^−2^ and energy density of 27.1 µWh cm^−2^. Microfluidic‐directed nitrogen‐doped graphene with both large SSA and uniform porous structure facilitated ions with fast diffusion and accumulation, resulting in a promising electrochemical performance (energy density is 95.7 µWh cm^−2^).^20^ The dot‐sheet (carbon dots/graphene)^22^ and core‐sheath (polyaniline/graphene)^23^ structures were also constructed by microfluidic method, which gave the SCs large electric double layer capacitance (EDLC) and pseudocapacitance. Other attempts have been developed to introduce electroactive materials (e.g., metal oxides/sulfides (MoS_2_,^24^ Co_3_O_4_,^25^ MnO_2_ 26), conducting polymer (polyaniline,^23^ polypyrrole^27^), and heteroatoms doping (N, S, P)^28^) in fibers to improve the energy storage abilities by redox and quantum capacitances. Though those approaches have enhanced the energy densities of FMSCs, two important issues should be addressed urgently: (1) The solid fibers can only use outer surface, whereas the compact internal microstructure with less porosity reduces ionic transport channels and accessible surface areas. (2) Poor charge transfer from active materials to conductive carbon materials decreases the active materials utilization, electrical conductivity, and redox reaction ability.

In this work, we develop a novel microfluidic strategy to design ordered porous core–shell fibers in which the core part is uniform porous graphene fiber (P‐GF) and the shell part is the electrochemical active material of vertically aligned NiO nanosheets (VA‐NiONSs). Through homogeneous microfluidic assembly, the core graphene fibers exhibit uniformly porous structure both in internal and external areas, which facilitates faster ion transportation kinetics. Additionally, the in situ vertical growth of nickel oxide arrays on graphene fiber guarantees the high charge transfer, promoting the sufficient redox reaction process of active materials. Due to microfluidic‐architected core–shell fibers with plentiful ionic channels, large SSA of 425.6 m^2^ g^−1^, higher electrical conductivity of 176.6 S cm^−1^, redox activity, and mechanical strength, FMSCs display a large specific capacitance of 605.9 mF cm^−2^, ultrahigh energy density of 120.3 µWh cm^−2^, and excellent cycling stability (95.1% of initial capacitance retention after 10 000 cycles). Based on those impressive properties, FMSCs woven into textiles can stably light up smart watch and light‐emitting diodes (LEDs). More specially, a self‐powered device, integrating solar cell and FMSCs into fabric can significantly power displays. Our study highlights the microfluidic method to synthesize the advanced structural materials in flexible energy‐storage technique as well as guides the progress of new‐generation portable and wearable electronics.

## 2 Results

### 2.1 Microfluidic Synthesis of Ordered Core–Shell‐Structured VA‐NiONSs/P‐GF

**Figure 1:**
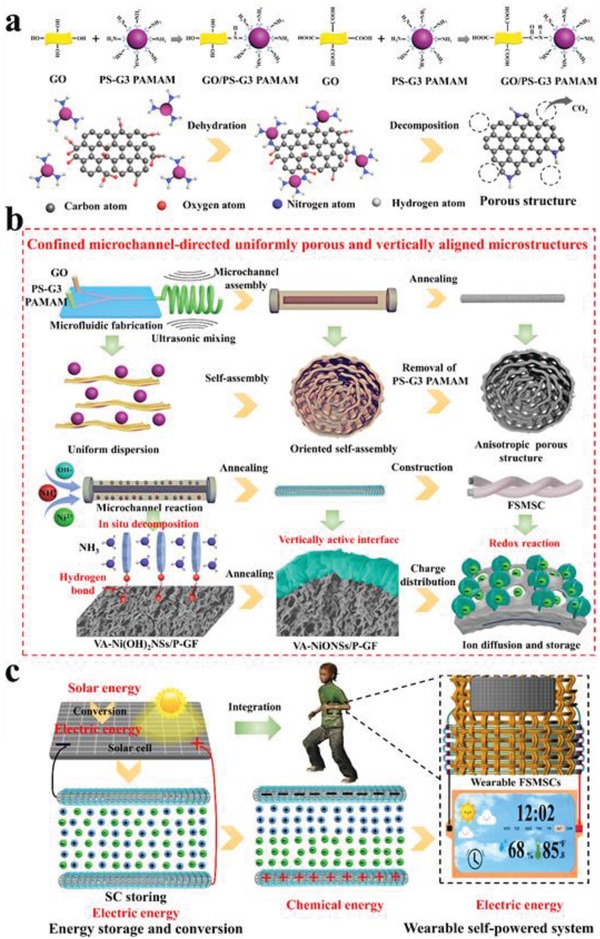
Schematic illustration of the microfluidic synthesis of VA‐NiONSs/P‐GF. a) The bonded mechanism of between PS‐G3 PAMAM and GO. b) Microfluidic fabrication of core–shell fiber and the construction of FMSCs. c) The FMSCs woven into textile to power electronics.

Advanced structure and activity of electrode materials, enabling faster electron conduction and ion diffusion kinetics are essential for achieving high energy density of FMSCs.^29^ To this end, we propose a microfluidic method that precisely controls the structure, morphology, and composition^30^ to fabricate orderly core–shell‐structured fiber electrodes (**Figure** **1**). To design the uniformly porous structure of fiber both in internal and external areas, we used monodispersed generation 3 polyamidoamine (G3 PAMAM) dendrimer‐coated polystyrene (PS) with a diameter around 85 nm (Figures S1–S3, Supporting Information) as template. Because the G3 PAMAM dendrimer (Figure S4, Supporting Information) is abundant with amino groups, the PS‐G3 PAMAM could be interacted with graphene oxide (GO) through dehydration condensation (Figure 1a). Once the template was removed, the uniform porous graphene fiber was obtained. Figure 1b schematically illustrates the microfluidic fabrication of core–shell‐structured fibers. First, a Y‐shaped microchip device (Figure S5, Supporting Information) with two channels featuring one flow of GO and the other flow of PS‐G3 PAMAM was developed. The two flows were evenly mixed to form well dispersion through intensive sonication (Figure S6, Supporting Information). Next, the well‐dispersion was hydrothermally confined self‐assembled to generate reduced‐GO nanosheets/ PS‐G3 PAMAM crosslinked composite fibers. At this stage, the PS‐G3 PAMAM with –NH_2_ group was bonded with the rich oxygen functional groups (e.g., carboxyl and hydroxyl) of GO. Additionally, the inherent liquid crystal characteristic of GO allowed the orderly aligned assembly, endowing the fiber with anisotropic structure.^31^ Then, after high‐temperature annealing, the PS‐G3 PAMAM was thermally decomposed, which could be verified by thermogravimetric analysis (TGA) (Figure S7, Supporting Information). Thereby, the uniform porous and anisotropic network of P‐GF with high electrical conductivity was achieved.

To make the fiber with high electrochemical activity, NiO nanomaterials were in situ deposited on P‐GF to form core–shell‐structured hybrid fiber via a microchannel reaction. It is worth mentioning that NiO materials are chosen primarily because of their adjustable microstructure and pseudo‐capacitance in energy storage field.^32^, ^33^ By immerging the P‐GF in inorganic salt solution (NiCl_2_, NH_4_Cl, and NaOH) under microreactor, the formed NH_3_ could be complex with Ni^2+^, which would further bridge with oxygen functional groups on P‐GF surface and produced sheet‐like Ni(OH)_2_ crystals.^34^, ^35^ Those sheets tended to be perpendicularly grown and cross‐linked with each other due to the decrease of surface energy.^34^ Over time, the well‐defined and vertically aligned Ni(OH)_2_ nanosheet arrays/P‐GF was obtained. After calcination, the core–shell‐structured VA‐NiONSs/P‐GF was achieved. Subsequently, the flexible solid‐state FMSC was constructed by the integration of two pieces of as‐fabricated hybrid fiber electrodes with polymer‐supported gel‐type electrolyte. Owing to the microfluidic assembly and reaction, the as‐fabricated hybrid fibers could be continuously synthesized with a length more than 0.5 m (Figure S8, Supporting Information), which is potential for large‐scale production. Meanwhile, the hybrid fibers exhibited super flexibility and weavability, which could be integrated into textiles withstanding consecutively bending deformation (Figure S9, Supporting Information). Due to the ordered porous structure, electrochemical activity and high mechanical properties of fibers, a proof‐of‐concept of wearable self‐powered system has been realized by making a combination of commercial solar cell and FMSCs woven into textile. This self‐powered device can not only harvest and convert solar energy to electric energy (solar cell unit) but also store electric energy (supercapacitor unit) to power displays (Figure 1c). Considering the microfluidic‐architected approach of core–shell fiber electrodes, we benefit many advantages from this design: (1) The homogeneous diffusion and assembly of GO and PS‐G3 PAMAM via microfluidic method guarantee the generated graphene fiber with uniformly porous network, large SSA, anisotropic structure and high electron conduction, accelerating ion with rapider diffusion and greater accumulation. (2) The microchannel confined reaction is particularly desirable to enable ordered NiO nanosheets with highly aligned 3D framework and abundant ionic pathways under an evenly vertical‐deposition on graphene fiber, which enhances the interfacial charge transfer, ion dynamic, and sufficient redox activity. (3) The uniform self‐assembly in microreactor allows the of composite fibers with striking mechanical flexibility, resulting in that the constructed solid‐state FMSCs are robust enough as energy supply to stably power electronic devices.

### 2.2 Microstructural Characterization of Ordered Core–Shell‐Structured VA‐NiONSs/P‐GF

**Figure 2:**
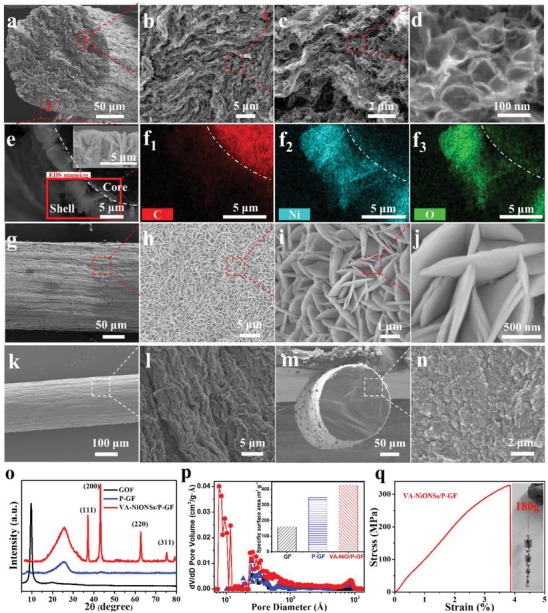
Structural characterization. a–d) Cross‐sectional SEM images of VA‐NiONSs/P‐GF at low, middle, and high magnifications, respectively. e) SEM images and f) EDS mapping for C, Ni, and O of VA‐NiONSs/P‐GF. g–j) Surface SEM images of VA‐NiONSs/P‐GF at low and high magnifications, respectively. k,l) Surface SEM images of pristine graphene fiber. m,n) Cross‐sectional SEM images of pristine graphene fiber. o) XRD patterns of as‐prepared samples. p) Pore size distributions of as‐prepared samples. q) Stress–strain curve of VA‐NiONSs/P‐GF.

The morphologies of core–shell‐structured fibers are analyzed by scanning electron microscopy (SEM). As illustrated from the cross‐sectional SEM image in **Figure** **2**a, the VA‐NiONSs/P‐GF displays an anisotropic backbone along the longitudinal direction with a diameter of 220 µm. The enlarged inner structures of VA‐NiONSs/P‐GF are shown in Figure 2b,c. Obviously, a highly porous and aligned structures are well‐interlinked inside fiber. Especially, those uniform pores corresponding to PS size of around 85 nm are clearly observed from high magnification SEM images (Figure 2d). It is revealed that those homogeneous pores can create a higher porosity for ion faster diffusion, and the EDLC of FMSCs will be increased.^36^ Additionally, regarding the observation at the interface of core–shell fiber, the NiO nanosheets with a height of around 5 µm are vertically covered on graphene fiber (Figure 2e). To investigate the combination between VA‐NiONSs and P‐GF, the energy dispersive spectroscopy (EDS) mapping for identifying the elements distribution is carried out. Obviously, the C element is mainly detected in the core parts of fiber, and Ni and O elements are well‐distributed on the shell areas (Figure 2f). It is noted that the NiO nanosheets are in situ grown on the graphene fiber, confirming the high charge transfer at the interface of hybrid fiber. Figure 2g–j shows the typical SEM images, illustrating the surface morphologies of VA‐NiONSs/P‐GF. Interestingly, the uniform NiO nanosheets with a thickness of 50 nm are aligned and densely deposited on the entire surface of graphene fiber. Undoubtedly, those NiO nanosheet arrays are well‐interconnected with each other to form highly continuous 3D networks. It is indicated that the regular array morphology and NiO composition have considerable effects on energy storage performance because the pseudo‐capacitances are mainly dominated by the rapid surface redox reaction for storing charges.^37^ However, for pristine graphene fiber, the densely wrinkled surface (Figure 2k,l) and compact cross section (Figure 2m,n) are preserved, which severely degrade the ionic diffusion pathways. It is found that the microfluidic method can provide a homogeneous atmosphere for precursors diffusion, assembly, and reaction, thereby this vertically aligned shell and orderly porous core are achieved.

The crystal phases of as‐prepared VA‐NiONSs/P‐GF, graphene oxide, and graphene fibers samples are characterized by the X‐ray diffraction (XRD) pattern (Figure 2o). The well‐defined diffraction peaks at 9.6° and 25.4° are assigned to the typical features of graphene oxide and graphene fibers,^31^, ^38^ demonstrating the thermal reduction of GO fiber. Strong diffraction peaks at around 37.1°, 43.2°, 62.8°, and 75.4° correspond to (110), (200), (220), and (311) characteristic planes of NiO cubic phase,^32^ respectively. Figure 2p shows the pore size distributions of as‐synthesized samples by N_2_ absorption–desorption isotherm measurement. Apparently, the VA‐NiONSs/P‐GF presents the widest pore size distribution in a range from micro‐, meso‐ to macropores (0.77–126.23 nm, particularly these abundant pores, 0.77–1.99 nm). By comparison, the porous graphene and pristine graphene fibers have narrower pore size distributions (porous graphene is 1.77–126.23 nm; pristine graphene is 2.52–126.23 nm). It is noted that one distinct pore peak at ≈87 nm in both VA‐NiONSs/P‐GF and P‐GF is attributed to the decomposition of PS. Benefiting from the predominated micro/mesopores, especially these measured values < 2 nm, the VA‐NiONSs/P‐GF preserves a significant larger SSA of 425.6 m^2^ g^−1^ than those of porous graphene (348.3 m^2^ g^−1^) and pristine graphene (159.4 m^2^ g^−1^) fibers. The mechanical strength of fibers is described by the stress–strain curve in Figure 2q. Admirably, the VA‐NiONSs/P‐GF fiber shows an excellent Young modulus of 122.3 MPa and breaking elongation of 3.83%, which can lift 180 g of weights. Therefore, concerning the fiber electrodes with ordered structure and electrochemical activity, this uniformly porous structure and vertical alignment of active interface are first realized by microfluidic method, which might tremendously boost electron transfer, ion diffusion, and redox process for improving energy densities of FMSCs.

### 2.3 Electrochemical Performances of VA‐NiONSs/P‐GF‐Based Solid‐State MSC

**Figure 3:**
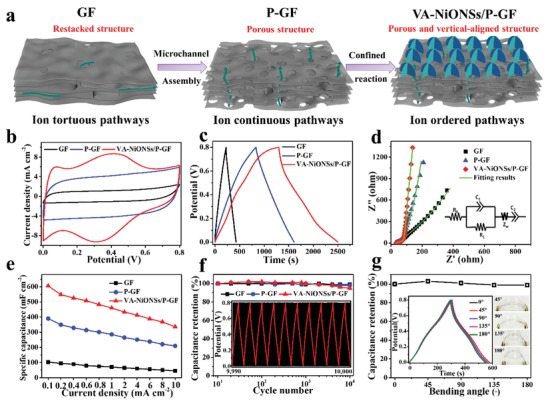
Electrochemical performances of solid‐state VA‐NiONSs/P‐GF MSC based on KOH/PVA gel electrolyte. a) Schematic diagram of designed GF, P‐GF, and VA‐NiONSs/P‐GF. b) CV curves of FMSCs at the scan rate of 20 mV s^−1^. c) GCD curves of FMSCs at a current density of 0.1 mA cm^−2^. d) EIS analysis of FMSCs. Inset: the equivalent circuit model. e) Calculated specific capacitances of FMSCs. f) Cyclic stability of FMSCs under continuous charge/discharge operation. Inset: GCD curves from 9990th to 10 000th cycles. g) Capacitance retentions of VA‐NiONSs/P‐GF based MSC under different bending angles (45°, 90°, 135°, and 180°) at the current density of 0.4 mA cm^−2^. Inset: GCD curves and pictures under different bending angles.

The practical FMSC was constructed by covering the gel‐state polyvinyl alcohol (PVA) supported KOH electrolyte on two pieces of VA‐NiONSs/P‐G fiber electrodes. The electrochemical performances of solid‐state FMSCs are tested by the cyclic voltammetry (CV) and galvanostatic charge/discharge (GCD) measurements. In our systems, three kinds of electrode materials are designed (**Figure** **3**a): restacked structure of pristine graphene fiber (GF), uniform porous structure of P‐GF, and orderly aligned structure of VA‐NiONSs/P‐GF. As shown from the comparison of CV cures in Figure 3b, the pristine graphene based MSC displays the smallest CV area with rectangular shape of EDLC, indicating the poor ion diffusion dynamic in electrode networks. After introducing the uniformly porous structure, the P‐GF presents a larger CV area than that of pristine graphene, which demonstrates the superior capacitive behavior and favorable charge transport in porous frameworks. Additionally, upon designing the ordered electroactive interface, the VA‐NiONSs/P‐GF obviously presents the largest integrated area with a couple of characteristic redox peaks, evidently demonstrating the best electrochemical performance and ion migration ability throughout the aligned electrode skeleton. This redox reaction derived from the Faradaic reactions of NiO nanosheets mainly corresponds to the reversible Ni^3+^/Ni^2+^ transitions, relating to electrolyte OH^−^ ions (NiO + OH^−^ ↔NiOOH + e^−^).^34^ Figure 3c and Figures S10–S12 (Supporting Information) illustrate the GCD behaviors of FMSCs. Outstandingly, the VA‐NiONSs/P‐GF maintains the best charge–discharge capability with nearly symmetric curve, confirming the fastest interfacial charge transfer, good reversibility, and high Coulombic efficiency.

In light of better understanding the ion diffusion kinetics in ordered structure‐based FMSCs, the electrochemical impedance spectroscopy (EIS) measurement is carried out.^39^ Figure 3d shows the Nyquist plots, including three parts: one depressed semicircle at the high‐frequencies, Warburg diffusion at the medium‐frequencies, and a vertical line at the low‐frequencies. The detailed element values are analyzed by the equivalent circuit mode (inset of Figure 3d): the parameters of *R*_0_ is the inner resistance of supercapacitor (mainly due to electrolyte); *R*_1_ is the charge transfer resistance (kinetic of electron transfer); *C*_1_ is the double‐layer capacitance; *Z*_w_ is Warburg impedance (kinetic of ion diffusion); *C*_2_ is intercalation capacitance (structures and redox reaction). Based on the fitted results in Table S1 (Supporting Information), the *R*_0_ is almost at the same value, revealing the same construction and measurement processes of FMSCs. When regarding to and *C*_1_/*R*_1_, a slight increase in the impedance of VA‐NiONSs/P‐GF (0.38 mF/4.67 Ω) compared with P‐GF (0.45 mF/3.99 Ω) and pristine graphene (0.47 mF/3.52 Ω) confirms the impressive charge transfer. It is because of the single nature of NiO nanosheets with high electron mobility that the excellent charge transfer from arrays to porous graphene is obtained. Considering the ion diffusion ability of *Z*_w_, the VA‐NiONSs/P‐GF preserves the lowest resistance of 47.2 Ω than those of P‐GF (69.3 Ω) and pristine graphene (295 Ω). It is indicated that the VA‐NiONSs/P‐GF possesses externally vertically aligned arrays and internal uniform porous for facilitating ion rapid diffusion. Moreover, in the low frequency regions, the sloped line of VA‐NiONSs/P‐GF is closer to the theoretically vertical line, implying a better capacitive behavior. As a result, the largest intercalation capacitance of VA‐NiONSs/P‐GF (0.053 F) is realized than those of P‐GF (0.035 F) and pristine graphene (0.008 F), which must be due to the pseudo‐capacitance and vertically aligned structure by ordered NiO arrays interface. Therefore, those results demonstrate that this designed VA‐NiONSs/P‐GF with uniform porous network and aligned active interface is beneficial for electron conduction and ion diffusion, bringing about a high energy‐storage performance.

The specific areal capacitances of FMSCs calculated from discharge times under different densities are described in Figure 3e. Significantly, the VA‐NiONSs/P‐GF displays a large capacitance of 605.9 mF cm^−2^, which is 587% and 155% higher than those of pristine graphene (103.2 mF cm^−2^) and P‐GF (390.4 mF cm^−2^), respectively at current density of 0.1 mA cm^−2^. To our knowledge, this levels is larger than most of currently reported carbon‐fibers based MSCs (graphene^9^ of 1.7 mF cm^−2^, CNTs/mesoporous carbon^18^ of 39.7 mF cm^−2^, CNT twisted fiber^40^ of 92.1 mF cm^−2^, graphene/Ni/Cu^10^ of 133 mF cm^−2^, CNT/PEDOT^41^ of 164.8 mF cm^−2^, graphene/CNTs^8^ of 177 mF cm^−2^, graphene/PANI^23^ of 230 mF cm^−2^, hollow graphene/PEDOT:PSS^21^ of 304.5 mF cm^−2^, and MnO*_x_*@TiN nanowires@CNTs^42^ of 360 mF cm^−2^). Additionally, the VA‐NiONSs/P‐GF can remain a good capacitance of 336.2 mF cm^−2^ even at such a higher density of 10 mA cm^−2^, whereas P‐GF and pristine graphene only preserve 203.6 and 24.8 mF cm^−2^, respectively. The long‐term cycling stability of FMSC, an important parameter evaluating the practical application is determined in Figure 3f. Satisfactorily, the VA‐NiONSs/P‐GF exhibits a steadily continuous charge/discharge process for 10 000 cycles without any noticeable degradation, which maintains a capacitance retention of 95.1%. To further investigate the flexibility of FMSC, the electrochemical performances under different bending angles (45°, 90°, 135°, and 180°) are conducted. As shown in Figure 3g, no obvious capacitance losses of charge/discharge curves are detected when withstanding bending deformations, implying the super durability of FMSC. Meanwhile, the device is robust enough to undergo repeated 180° bending deformation for 1000 times (Figure S13, Supporting Information). The greatly cyclic stability and deformable durability are attributed to the in situ growth of core–shell structure and crystalline feature of VA‐NiONSs/P‐GF, along with good flexibility and mechanical strength.

### 2.4 Interfacial Nanostructure Analysis of VA‐NiONSs/P‐GF MSC

**Figure 4:**
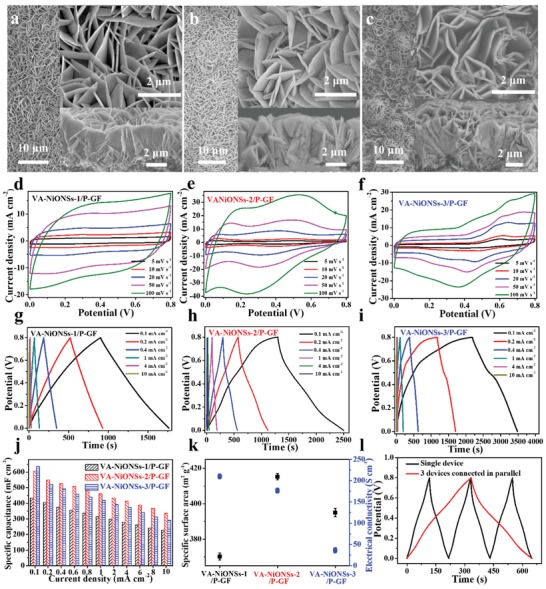
Interfacial nanostructure analysis. a–c) Surface and cross‐sectional SEM images of VA‐NiONSs‐1/P‐GF, VA‐NiONSs‐2/P‐GF, and VA‐NiONSs‐3/P‐GF, respectively. d–f) CV curves of VA‐NiONSs‐1/P‐GF, VA‐NiONSs‐2/P‐GF, and VA‐NiONSs‐3/P‐GF at different scan rates, respectively. g–i) GCD curves of VA‐NiONSs‐1/P‐GF, VA‐NiONSs‐2/P‐GF, and VA‐NiONSs‐3/P‐GF at different current densities, respectively. j) Calculated specific capacitances of VA‐NiONSs‐1/P‐GF, VA‐NiONSs‐2/P‐GF, and VA‐NiONSs‐3/P‐GF, respectively. k) Relationship between SSA and electrical conductivity of VA‐NiONSs‐1/P‐GF, VA‐NiONSs‐2/P‐GF, and VA‐NiONSs‐3/P‐GF. l) GCD curves of single and three FMSCs connected in parallel.

Because the pseudo‐capacitances are mainly governed by the interfacial aligned NiO nanosheets, we investigate the heights and densities of NiO arrays by tailoring the chemically deposited times (1 h for VA‐NiONSs‐1/P‐GF, 2 h for VA‐NiONSs‐2/P‐GF, and 3 h for VA‐NiONSs‐3/P‐GF). **Figure** **4**a shows that the VA‐NiONSs‐1/P‐GF presents a sparse nanosheets distribution with a height of 3.2 µm and thickness of 26 nm. The height and thickness of NiO nanosheets for VA‐NiONSs‐2/P‐GF (Figure 4b) are increased to 5 µm and 50 nm upon prolonging the deposition time. However, when further increasing the growth time, the NiO nanosheets will be over grown, stacked, and crumpled to form microspheres with a height of 6.8 µm for VA‐NiONSs‐3/P‐GF (Figure 4c), which might block the porosity of nanomaterials.^32^ The CV profiles, clarifying the redox reaction on NiO nanosheet surfaces at different scan rates are shown in Figure 4d–f. Exceptionally, the VA‐NiONSs‐2/P‐GF displays obvious redox peaks with large areas than those of VA‐NiONSs‐1/P‐GF and VA‐NiONSs‐3/P‐GF at the whole scan rates, demonstrating the reversible redox reaction and high charge transfer process on NiO arrays.

Figure 4g,h shows the nearly symmetric GCD curves for VA‐NiONSs‐1/P‐GF and VA‐NiONSs‐2/P‐GF, indicating the highly reversible ability, whereas the VA‐NiONSs‐3/P‐GF presents a much longer charge time than that of discharge time (Figure 4i). It is implied that the overgrowth of NiO nanosheets deteriorate the ion diffusion process and rate capability. Due to the excessive deposition of NiO nanosheets, the NiONSs‐3/P‐GF exhibits the largest specific capacitance of 635 mF cm^−2^ at a current density of 0.1 mA cm^−2^ (Figure 4j). However, when raising the current densities from 0.2 to 10 mA cm^−2^, the capacitances decrease severely. Notably, over an entire range of current densities, the VA‐NiONSs‐2/P‐GF basically maintains the best capacitance retentions. Those results must be associated with the intrinsic properties of fibers. Thus, we further evaluate the SSA and electrical conductivity of fibers. As illustrated in Figure 4k and Figure S14 (Supporting Information), comparing VA‐NiONSs‐2/P‐GF with VA‐NiONSs‐1/P‐GF, a slightly decrease of electrical conductivity in VA‐NiONSs‐2/P‐GF (176.7 S cm^−1^) significantly enhances the SSA (425.6 m^2^ g^−1^) in contrast to VA‐NiONSs‐1/P‐GF (electrical conductivity of 210.1 S cm^−1^, SSA of 370.2 m^2^ g^−1^). For VA‐NiONSs‐3/P‐GF, the overgrowth of NiO reduces both electrical conductivity (36.2 S cm^−1^) and SSA (395 m^2^ g^−1^). In particular, the stacking NiO nanosheets decrease the ion absorption surface area and porosity between electrode and electrolyte interface, which leads to the severe decline of capacitances at high current densities. Therefore, by balancing the electrical conductivity and SSA, the VA‐NiONSs‐2/P‐GF with the optimal height and density has the best capacitance and rate capability.

For satisfying the practical application of high energy and power needs, we integrate the FMSCs in series and parallel to realize higher current and voltage outputs. Figure 4l and Figure S15 (Supporting Information) show that three FMSCs are connected in parallel. The corresponding discharge time reaches three times higher than that of single FMSC at the same applied voltage, indicating that the output current is elevated. Additionally, by assembling three FMSCs in series, the operating voltage is increased from 0.8 to 2.4 V under almost the same discharge time (Figure S16, Supporting Information). Thus, the integration of FMSCs in series and parallel to control the output current and voltage further confirms the stability of designed core–shell structure and single crystalline NiO nature.

### 2.5 Electrochemical Performances of VA‐NiONSs/P‐GF‐Based High‐Energy MSC and Wearable Application

**Figure 5:**
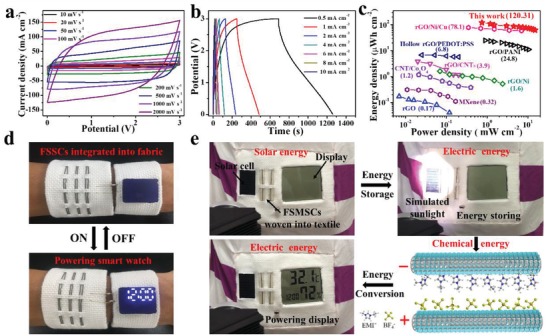
Electrochemical performances of solid‐state VA‐NiONSs/P‐GF MSC based on EMIMBF4/PVDF‐HFP gel electrolyte. a) CV curves of MSC at different scan rates. b) GCD curves of MSC at different current densities. c) Areal energy density and power density of VA‐NiONSs/P‐GF MSC and its comparison with nanocarbon electrode‐based MSCs. d) FMSCs integrated into textile as energy supplies to power smart watch. e) Photographs of FMSCs assembled with solar cell to power display.

Increasing the operating voltage is an effective way to achieve high energy density (*E* = 1/2 *CV* ^2^).^36^ In this regard, the poly(vinylidene fluoride‐*co*‐hexafluoropropylene) (PVDF‐HFP) supported 1‐eutyl‐3‐methylimidazolium tetrafluoroborate (EMIMBF_4_) ionic liquid is ideal used because of its fascinating characteristics of wider operating voltage window, negligible vapor pressure, and high ionic conductivity.^43^ Hence, the FMSCs can be measured in a larger electrochemical window of 0–3 V. As described in **Figure** **5**a, the CV curves of VA‐NiONSs/P‐GF maintain the rectangular shapes with no obvious redox peaks at different scan rates of 10–2000 mV s^−1^. Even at such a high scan rate of 2000 mV s^−1^, a nearly rectangular shape can still be retained, demonstrating the ideal capacitive characteristic and faster charge/discharge ability of fiber. Figure 5b is the typical GCD curves of VA‐NiONSs/P‐GF under current densities in the range of 0.5–10 mA cm^−2^. The almost linear and symmetric triangular shapes imply the good reversibility and charge propagation across the fiber electrodes. The columbic efficiencies of VA‐NiONSs/P‐GF supercapacitor based on EMIMBF_4_/PVDF‐HFP gel electrolyte layer are 87.5%, 91.6%, and 97.2% at current densities of 0.5, 1, and 10 mA cm^−2^, respectively. It is revealed that VA‐NiONSs/P‐GF provides the ordered and porous pathways for ion rapid diffusion. Considering those features, the VA‐NiONSs/P‐GF based MSC exhibits a large areal capacitance of 385 mF cm^−2^ at a current density of 0.5 mA cm^−2^ (Figure S17, Supporting Information). Furthermore, the energy density and power density are key important parameters for determining the practical application of FMSCs (Figure 5c). Remarkably, the highest real energy density of 120.3 µWh cm^−2^ is obtained at a power density of 0.75 mW cm^−2^ and it can still keep 62.2 µWh cm^−2^ even at a particularly high power density of 15 mW cm^−2^. To our knowledge, the energy density value of our fiber‐based MSC is evidently one of the highest levels among previously reported FMSCs, as illustrated in Table S2 (Supporting Information) (graphene^9^ of 0.17 µWh cm^−2^, MXene^1^ of 0.32 µWh cm^−2^, CNTs/Co_3_O_4_ 44 of 1.2 µWh cm^−2^, graphene/Ni^45^ of 1.6 µWh cm^−2^, graphene/CNTs^8^ of 3.9 µWh cm^−2^, hollow graphene/PEDOT:PSS^21^ of 6.8 µWh cm^−2^, graphene/PANI^23^ of 24.8 µWh cm^−2^, graphene/carbon dots of 67.37^22^ µWh cm^−2^, graphene/Ni/Cu^10^ of 78.1 µWh cm^−2^).

To confirm this high‐energy‐storage device for meeting practically wearable application, the FMSCs are integrated into flexible and textile substrates as energy supplies to power electronic devices. As shown in Figure S18 (Supporting Information), FMSCs are integrated in parallel to stably light up ten LEDs. Additionally, five FMSCs assembled in flexible substrates can power time‐meter and clock (Figures S19 and S20, Movies S1 and S2, Supporting Information). Meanwhile, three MSCs assembled into textile to power wearable smart watch (Figure 5d). More specially, a proof‐of‐concept of wearable self‐powered device has been designed by combining the commercial solar cell and FMSCs. Upon illuminating the simulated sunlight, the solar cell can harvest and convert solar energy into electric energy, which can be further stored by wearable FMSCs (Figure 5e; Figure S21 and Movie S3, Supporting Information). As a result of consecutive conversion and storage, this self‐charged device can outstandingly power display, which will open up a promising potential for new‐energy storage technology. Therefore, considering those remarkable electrochemical performance together with practical application, the VA‐NiONSs/P‐GF with ordered porous network, aligned interface and large SSA might become a prime candidate to bridge the gap between flexible MSCs and microbatteries and even substitute for microbatteries in future wearable industry.

## 3 Discussion

**Figure 6:**
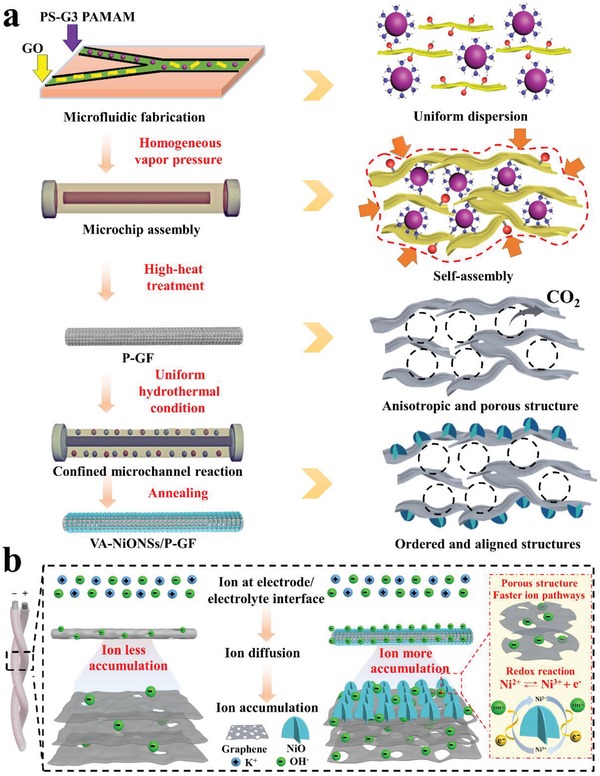
a) Scheme of microfluidic‐architected ordered porous and vertically aligned structures. b) Mechanism for VA‐NiONSs/P‐GF based MSC.

In light of comprehensively analyzing the results, the high‐performance mechanisms of FMSCs are illustrated in **Figure** **6**. The outstandingly electrochemical performances of FMSCs are primarily originated from the microfluidic‐architected internally uniform porous nanostructure and externally aligned interface of fiber electrodes. As described in Figure 6a, when injecting GO and PS‐G3 PAMAM in microchip with ultrasonic mixing process, the microfluidic fabrication creates a homogeneous vapor pressure for precursors' even dispersion, diffusion, and assembly. The uniform reaction greatly boosts the –NH_2_ groups in PS‐G3 PAMAM template bonding with oxygen functional groups in GO. After removing template, the uniform porous networks with large accessible surface area are generated for ions with faster migration and accommodation. Meanwhile, the microchannel can largely induce the GO liquid crystals to assemble into anisotropic structure, further accelerating ions motion. Additionally, by manipulating the chemical reaction in confined microchannel, the well‐defined nanosheet arrays of NiO are vertically grown on P‐GF, which guarantees the high charge transfer from conductive graphene to active material. It is the due to the microfluidic‐architected uniformly porous network, anisotropic structure, and vertically aligned active interface that the FMSCs exhibit higher energy‐storage performances.

The detailed mechanism is shown in Figure 6b. For pristine graphene, due to the densely compacted structure, the mainly utilized outer regions of fiber with less pores and accessible SSA (159.4 m^2^ g^−1^) cause ions with slower diffusion and less accumulation into restacked networks. Thus, the pristine graphene performs the smallest EDLC. However, by manipulating well‐defined porous structure in fiber, the porosity and SSA have been greatly improved for 218%. Sufficient path channels (micro‐ and mesopores) are created to minimize ionic diffusion distances and make ion rapid motion and local accommodation^3^, ^46^ so that EDLC is significantly enhanced. Additionally, by designing the vertically aligned NiO arrays, the ordered interface has further developed the micropores and SSA (267% improvement). It is indicated that those pores below 1 nm are beneficial to the contribution of capacitance because they are closer to ion size.^47^ More importantly, the in situ deposited crystalline NiO nanosheets on P‐GF with high electron mobility ensure the ideal interfacial charge transfer and electrolyte accessibility, which can efficiently boost the redox reaction and utilization of active materials. Particularly, the vertically oriented nanoarrays can also enable ions with quick diffusion. As a result, the VA‐NiONSs/P‐GF networks are favorable for ion smooth migration and waterfall‐like accumulation, leading to a high energy‐storage ability. Therefore, the microfluidic‐architected VA‐NiONSs/P‐GF with ordered porous and anisotropic alignment is vital to achieve larger electrochemical performance.

In summary, we have demonstrated a microfluidic strategy to design ordered structural and electrochemically active core–shell fibers. Underlying the homogeneous self‐assembly and reaction in microreactor, the graphene fibers exhibit an anisotropic backbone with uniform porous structure throughout the entire electrodes. The NiO arrays with well‐defined nanosheets are deposited vertically on graphene, guaranteeing a favorable charge transfer. As a result, the VA‐NiONSs/P‐GF has plentiful ionic pathways, large SSA (425.6 m^2^ g^−1^), high electrical conductivity (176.6 S cm^−1^), sufficient redox activity, and mechanical properties, which considerably facilitate ions with faster transportation and accommodation. The constructed FMSCs exhibit higher electrochemical performances, including large specific capacitance, higher energy density, excellent cycling stability, and stably deformable energy supply durability. Regarding those satisfactory merits, FMSCs can be integrated and woven into flexible and textile substrates to light up LEDs and smart watch. More importantly, a proof‐of‐concept of self‐powered system that converts and stores solar energy into electric energy has been designed to impressively power displays. This microfluidic strategy provides a new way to design advanced electrodes with both structural and active features, which will significantly promote the development of next‐generation miniaturized wearable electronics.

## 4 Experimental Section

*Materials*: Styrene (St), sodium hydroxide (NaOH), sodium bicarbonate (NaHCO_3_), sodium dodecyl sulfate (SDS), potassium persulfate (KPS), nickel chloride (NiCl_2_), methyl methacrylate (MMA), acrylic acid (AA), *N*‐(3‐dimethylaminopropyl)‐*N′*‐ethylcarbodiimide hydrochloride (EDC), *N*‐hydroxysuccinimide (NHS), ammonium chloride (NH_4_Cl), and phosphoric acid (H_3_PO_4_) were obtained from China Pharmaceutical Chemical Reagent Co., Ltd. Polytetrafluoroethylene (PTFE) tubes were purchased from Shanghai Li Quan Rubber & Plastic Co., Ltd. Aniline (ANI). PVA and PVDF‐HFP were achieved from Sigma‐Aldrich. EMIMBF_4_ (1‐eutyl‐3‐methylimidazolium tetrafluoroborate) was bought from Shanghai Chen Jie Chemical Reagent Co., Ltd.

*Synthesis of PS‐G3 PAMAM Latex*: SDS (0.05 g) and sodium bicarbonate (0.1 g) were added to four‐necked flask with 130 mL deionized water in oil bath at 85 °C under continuously stirring. Then, 5 g styrene (St) was slowly dripped into the flask until the solution became transparent and followed by chemical reaction for 1.5 h under high purity nitrogen gas protection. After that, 0.3 g MMA, 0.3 g AA, and 0.05 g KPS were added into the flask, which was kept for another 4 h at 90 °C. The obtained PS latex was filtered and washed by the deionized water for three times. Besides, 0.152 g of EDC in 2 mL deionized water and 0.075 g of NHS in 2 mL deionized water were added dropwise into the as‐prepared PS latex. After stirring for 1 h, 0.15 g G3 PAMAM dendrimers^48^ dispersed in 6 mL water were further added into the mixture, which was reacted for 24 h. Finally, the PS‐G3 PAMAM latex was obtained.

*Preparation of VA‐NiONSs/P‐GF*: GO was synthesized by oxidation of graphite powder based on a modified Hummers method.^49^ First, a Y‐shaped microchip device with two channels featuring core flow of GO (12 mg mL^−1^) and shell flow of PS‐G3 PAMAM (5 wt%) was developed. Then, the core flow (35 mL h^−1^) and shell flow (15 mL h^−1^) were injected into microchip by syringe pump, which were further evenly mixed to form well dispersion through intensive sonication. Afterwards, the microchip was sealed and undergone a hydrothermally (180 °C for 2h) confined self‐assembly to generate reduced‐GO nanosheets/PS‐G3 PAMAM crosslinked composite fibers after drying. Next, under Ar protection, the composite fibers were further heated up to 1000 °C (heating rate was 2.5 °C min^−1^) and maintained at 1000 °C for 2 h. After naturally cooling down at ambient temperature, P‐GF with uniform porous network was obtained. Besides, the P‐GF was further placed into NiCl_2_, NH_4_Cl, and NaOH solution (molar rate of NiCl_2_, NH_4_Cl, and NaOH is 1.15:6:2), which was chemically deposited to form vertically aligned Ni(OH)_2_/P‐GF hybrid fiber in a microreactor at 150 °C. After calcination at 400 °C for 2 h, the core–shell‐structured VA‐NiONSs/P‐GF was achieved. Pristine graphene fiber was fabricated by the same process.

*Construction of Wearable Micro‐SCs and Electrochemical Characterization*: The micro‐SC was constructed by covering gel‐like solid‐state electrolyte on two fiber electrodes. In this work, two kinds of solid‐state electrolytes (H_3_PO_4_/PVA and EMIMBF_4_/PVDF‐HFP) were used. For H_3_PO_4_/PVA electrolyte, 1 g H_3_PO_4_ and 1 g PVA were added into 10 mL deionized water, which was heated at 80 °C until it became clear. For EMIMBF_4_/PVDF‐HFP organic electrolyte, 2 g EMIMBF_4_ and 3 g PVDF‐HFP were added in 15 mL DMF solution, which was heated at 60 °C for 4 h.

For supercapacitors, CHI760E electrochemical work station was used to characterize CV, galvanostatic charge/discharge, and EIS performances. The specific areal capacitances of supercapacitor according to galvanostatic charge/discharge test was evaluated by the equation of *C*_A_ = $\frac{4I\Delta t}{A\Delta V}$, where *I*, Δ*t*, Δ*V*, and A (cm^2^) were the discharge current (A), discharge time (s), voltage range (V), and total area of two fiber electrodes, respectively. The energy density and power energy were calculated by equations of *E* = *C*_A_ *V* ^2^/8 and *P* = *E*/Δ*t*, where the *C*_A_, *V*, and Δ*t* were the areal capacitance, the operated voltage, and the discharge time. The columbic efficiency (CE) was calculated according to galvanostatic charge–discharge cycles by the equations of CE = Δ*t*_d_ */*Δ*t*_c_, where Δ*t*_d_ and Δ*t*_c_ represented the discharge time and charge time.

*The Fabrication Process of Self‐Powered System*: A wearable self‐powered system had been designed by the integration of the following components. Detailly, the self‐powered system consisted of four parts: solar cell, two FMSCs connected in parallel, display, and simulated sunlight. The commercial solar cell was used for harvesting and converting the solar energy into electrical energy, which had the operating voltage and current of 3 V and 80 mA. Two FMSCs connected in parallel were applied to store electrical energy, which can power the display. According to circuit assembly diagram in Figure S21 (Supporting Information), the solar cell was first connected with FMSCs, which was further integrated with display using a switch. Upon illuminating the simulated sunlight for several seconds, the solar cell could harvest and convert solar energy into electric energy, which was next stored by wearable FMSCs. As a result of consecutive conversion and storage, this self‐charged device could successfully power display.

## Conflict of Interest

The authors declare no conflict of interest.

## Supporting information

Supporting Information

Supplemental Video 1

Supplemental Video 2

Supplemental Video 3

## References

[1] C. J. Zhang , L. McKeon , M. P. Kremer , S.‐H. Park , O. Ronan , A. Seral‐Ascaso , S. Barwich , C. Ó. Coileáin , N. McEvoy , H. C. Nerl , Nat. Commun. 2019, 10, 1795. 30996224 10.1038/s41467-019-09398-1 PMC6470171

[2] X. Wu , Y. Xu , Y. Hu , G. Wu , H. Cheng , Q. Yu , K. Zhang , W. Chen , S. Chen , Nat. Commun. 2018, 9, 4573. 30385751 10.1038/s41467-018-06914-7 PMC6212570

[3] D. Yu , K. Goh , H. Wang , L. Wei , W. Jiang , Q. Zhang , L. Dai , Y. Chen , Nat. Nanotechnol. 2014, 9, 555. 24813695 10.1038/nnano.2014.93

[4] P. Simon , Y. Gogotsi , Nat. Mater. 2008, 7, 845. 18956000 10.1038/nmat2297

[5] a) P. Huang , C. Lethien , S. Pinaud , K. Brousse , R. Laloo , V. Turq , M. Respaud , A. Demortiere , B. Daffos , P.‐L. Taberna , Science 2016, 351, 691; 26912855 10.1126/science.aad3345

[6] T. Lv , M. Liu , D. Zhu , L. Gan , T. Chen , Adv. Mater. 2018, 30, 1705489. 10.1002/adma.201705489 29479744

[7] A. Sumboja , J. Liu , W. G. Zheng , Y. Zong , H. Zhang , Z. Liu , Chem. Soc. Rev. 2018, 47, 5919. 29947399 10.1039/c8cs00237a

[8] L. Kou , T. Huang , B. Zheng , Y. Han , X. Zhao , K. Gopalsamy , H. Sun , C. Gao , Nat. Commun. 2014, 5, 3754. 24786366 10.1038/ncomms4754 PMC4024741

[9] Y. Meng , Y. Zhao , C. Hu , H. Cheng , Y. Hu , Z. Zhang , G. Shi , L. Qu , Adv. Mater. 2013, 25, 2326. 23463634 10.1002/adma.201300132

[10] M. Liu , Z. Cong , X. Pu , W. Guo , T. Liu , M. Li , Y. Zhang , W. Hu , Z. L. Wang , Adv. Funct. Mater. 2019, 29, 1806298.

[11] a) G. Wu , X. Wu , Y. Xu , H. Cheng , J. Meng , Q. Yu , X. Shi , K. Zhang , W. Chen , S. Chen , Adv. Mater. 2019, 31, 1806492; 10.1002/adma.201806492 31012167

[12] A. S. Arico , P. Bruce , B. Scrosati , J. M. Tarascon , W. Van Schalkwijk , Nat. Mater. 2005, 4, 366. 15867920 10.1038/nmat1368

[13] F. Zhou , H. Huang , C. Xiao , S. Zheng , X. Shi , J. Qin , Q. Fu , X. Bao , X. Feng , K. Müllen , J. Am. Chem. Soc. 2018, 140, 8198. 29893575 10.1021/jacs.8b03235

[14] D. Pech , M. Brunet , P.‐L. Taberna , P. Simon , N. Fabre , F. Mesnilgrente , V. Conédéra , H. Durou , J. Power Sources 2010, 195, 1266.

[15] a) H. Durou , D. Pech , D. Colin , P. Simon , P.‐L. Taberna , M. Brunet , Microsyst. Technol. 2012, 18, 467;

[16] K. Shen , J. Ding , S. Yang , Adv. Energy Mater. 2018, 8, 1800408.

[17] Z. Liu , Z. S. Wu , S. Yang , R. Dong , X. Feng , K. Müllen , Adv. Mater. 2016, 28, 2217. 26784382 10.1002/adma.201505304

[18] J. Ren , W. Bai , G. Guan , Y. Zhang , H. Peng , Adv. Mater. 2013, 25, 5965. 23956005 10.1002/adma.201302498

[19] a) N. A. Jose , H. C. Zeng , A. A. Lapkin , Nat. Commun. 2018, 9, 4913; 30464298 10.1038/s41467-018-07395-4 PMC6249219

[20] G. Wu , P. Tan , X. Wu , L. Peng , H. Cheng , C. F. Wang , W. Chen , Z. Yu , S. Chen , Adv. Funct. Mater. 2017, 27, 1702493.

[21] G. Qu , J. Cheng , X. Li , D. Yuan , P. Chen , X. Chen , B. Wang , H. Peng , Adv. Mater. 2016, 28, 3646. 27001216 10.1002/adma.201600689

[22] Q. Li , H. Cheng , X. Wu , C.‐F. Wang , G. Wu , S. Chen , J. Mater. Chem. A 2018, 6, 14112.

[23] X. Wu , G. Wu , P. Tan , H. Cheng , R. Hong , F. Wang , S. Chen , J. Mater. Chem. A 2018, 6, 8940.

[24] J. Liang , G. Zhu , C. Wang , Y. Wang , H. Zhu , Y. Hu , H. Lv , R. Chen , L. Ma , T. Chen , Z. Jin , J. Liu , Adv. Energy Mater. 2017, 7, 1601208.

[25] F. Su , X. Lv , M. Miao , Small 2015, 11, 854. 25277293 10.1002/smll.201401862

[26] J. Yu , W. Lu , J. P. Smith , K. S. Booksh , L. Meng , Y. Huang , Q. Li , J.‐H. Byun , Y. Oh , Y. Yan , T.‐W. Chou , Adv. Energy Mater. 2017, 7, 1600976.

[27] A. Razaq , L. Nyholm , M. Sjödin , M. Strømme , A. Mihranyan , Adv. Energy Mater. 2012, 2, 445.

[28] a) Y. Liu , Y. T. Shen , L. T. Sun , J. C. Li , C. Liu , W. C. Ren , F. Li , L. B. Gao , J. Chen , F. C. Liu , Y. Y. Sun , N. J. Tang , H. M. Cheng , Y. W. Du , Nat. Commun. 2016, 7, 9;

[29] H. Sun , L. Mei , J. Liang , Z. Zhao , C. Lee , H. Fei , M. Ding , J. Lau , M. Li , C. Wang , Science 2017, 356, 599. 28495745 10.1126/science.aam5852

[30] a) L. L. Xu , C. F. Wang , S. Chen , Angew. Chem., Int. Ed. 2014, 53, 3988; 10.1002/anie.201310977 24595996

[31] G. Xin , T. Yao , H. Sun , S. M. Scott , D. Shao , G. Wang , J. Lian , Science 2015, 349, 1083. 26339027 10.1126/science.aaa6502

[32] G. Wu , G. Li , T. Lan , Y. Hu , Q. Li , T. Zhang , W. Chen , J. Mater. Chem. A 2014, 2, 16836.

[33] H. Zhang , X. Yu , P. V. Braun , Nat. Nanotechnol. 2011, 6, 277. 21423184 10.1038/nnano.2011.38

[34] C. Yuan , X. Zhang , L. Su , B. Gao , L. Shen , J. Mater. Chem. 2009, 19, 5772.

[35] Y. Zou , Y. Wang , Nanoscale 2011, 3, 2615. 21523266 10.1039/c1nr10070j

[36] Y. Xu , Z. Lin , X. Zhong , X. Huang , N. O. Weiss , Y. Huang , X. Duan , Nat. Commun. 2014, 5, 4554. 25105994 10.1038/ncomms5554

[37] a) G. Zhou , D.‐W. Wang , L.‐C. Yin , N. Li , F. Li , H.‐M. Cheng , ACS Nano 2012, 6, 3214; 22424545 10.1021/nn300098m

[38] X. Yang , C. Cheng , Y. Wang , L. Qiu , D. Li , Science 2013, 341, 534. 23908233 10.1126/science.1239089

[39] a) J. Bisquert , Electrochim. Acta 2002, 47, 2435;

[40] W. Son , S. Chun , J. M. Lee , Y. Lee , J. Park , D. Suh , D. W. Lee , H. Jung , Y.‐J. Kim , Y. Kim , Nat. Commun. 2019, 10, 426. 30683872 10.1038/s41467-018-08016-w PMC6347621

[41] X. Li , J. Shao , S.‐K. Kim , C. Yao , J. Wang , Y.‐R. Miao , Q. Zheng , P. Sun , R. Zhang , P. V. Braun , Nat. Commun. 2018, 9, 2578. 29968704 10.1038/s41467-018-04937-8 PMC6030180

[42] Z. Pan , J. Yang , Q. Zhang , M. Liu , Y. Hu , Z. Kou , N. Liu , X. Yang , X. Ding , H. Chen , Adv. Energy Mater. 2019, 9, 1802753.

[43] M. Armand , F. Endres , D. R. MacFarlane , H. Ohno , B. Scrosati , Nat. Mater. 2009, 8, 621. 19629083 10.1038/nmat2448

[44] Y. Zhu , S. Murali , M. D. Stoller , K. Ganesh , W. Cai , P. J. Ferreira , A. Pirkle , R. M. Wallace , K. A. Cychosz , M. Thommes , Science 2011, 332, 1537. 21566159 10.1126/science.1200770

[45] X. Pu , L. Li , M. Liu , C. Jiang , C. Du , Z. Zhao , W. Hu , Z. L. Wang , Adv. Mater. 2016, 28, 98. 26540288 10.1002/adma.201504403

[46] G. Wu , Y. Hu , Y. Liu , J. Zhao , X. Chen , V. Whoehling , C. Plesse , G. T. Nguyen , F. Vidal , W. Chen , Nat. Commun. 2015, 6, 7258. 26028354 10.1038/ncomms8258 PMC4458862

[47] a) J. Chmiola , G. Yushin , Y. Gogotsi , C. Portet , P. Simon , P.‐L. Taberna , Science 2006, 313, 1760; 16917025 10.1126/science.1132195

[48] G. Peng , Z. Zhu , Y. Tian , Y.‐l. Tong , T.‐T. Cui , C.‐F. Wang , S. Chen , J. Mater. Chem. C 2018, 6, 8187.

[49] D. Li , M. B. Müller , S. Gilje , R. B. Kaner , G. G. Wallace , Nat. Nanotechnol. 2008, 3, 101. 18654470 10.1038/nnano.2007.451

