# Supplementary material for: Microfluidic‐Architected Nanoarrays/Porous Core–Shell Fibers toward Robust Micro‐Energy‐Storage
Source: Adv Sci (Weinh). 2019 Nov 25;7(1):1901931. doi: 10.1002/advs.201901931 (PMC6947592; doi:10.1002/advs.201901931)
Supplement: Supplementary file 1 — Supporting Information [file ADVS-7-1901931-s001.pdf]

Copyright WILEY-VCH Verlag GmbH & Co. KGaA, 69469 Weinheim, Germany, 2019.

ADVANCED SCIENCE
Open Access

Supporting Information

for *Adv. Sci.,* DOI: 10.1002/advs.201901931

Microfluidic-Architected Nanoarrays/Porous Core–Shell Fibers toward Robust Micro-Energy-Storage

Jinku Meng, Guan Wu, Xingjiang Wu, Hengyang Cheng, Zhi Xu,* and Su Chen**

Copyright WILEY-VCH Verlag GmbH & Co. KGaA, 69469 Weinheim, Germany, 2019.

Supporting Information

Microfluidic-Architected Nanoarrays/Porous Core-Shell Fibers toward Robust Micro-Energy-Storage

Jinku Meng, Guan Wu, Xingjiang Wu, Hengyang Cheng, Zhi Xu* & Su Chen**

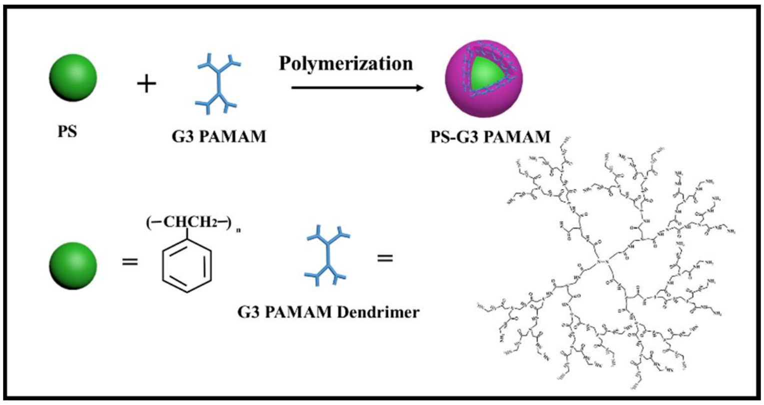

Figure S1. Scheme of preparation of PS-G3 PAMAM.

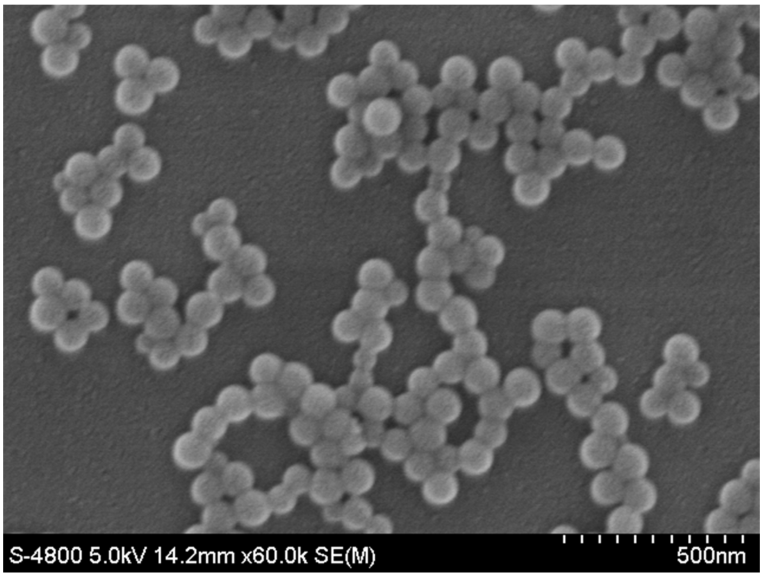

Figure S2. SEM of PS-G3 PAMAM.

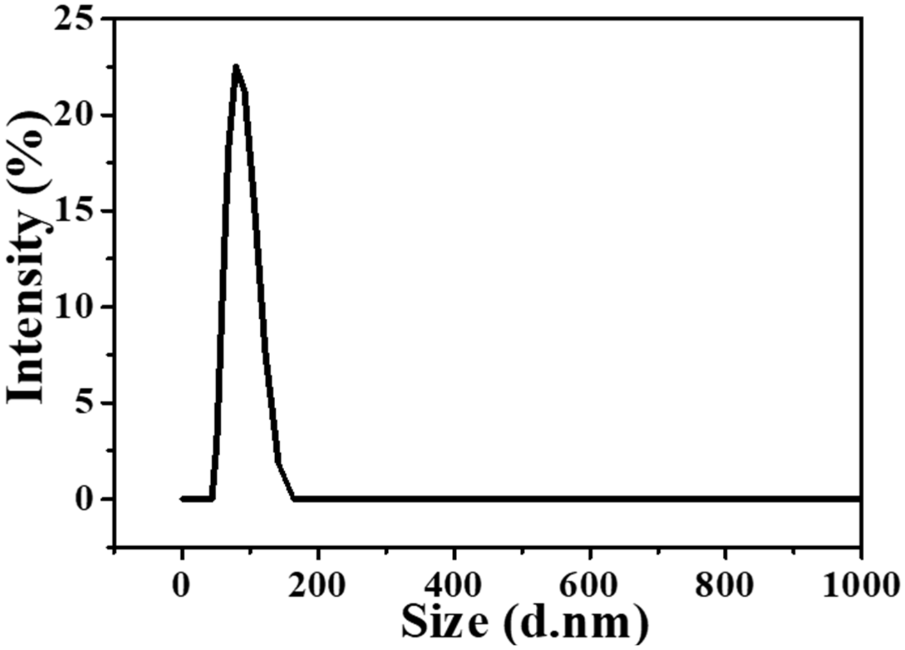

Figure S3. Particle size distribution of PS-G3 PAMAM

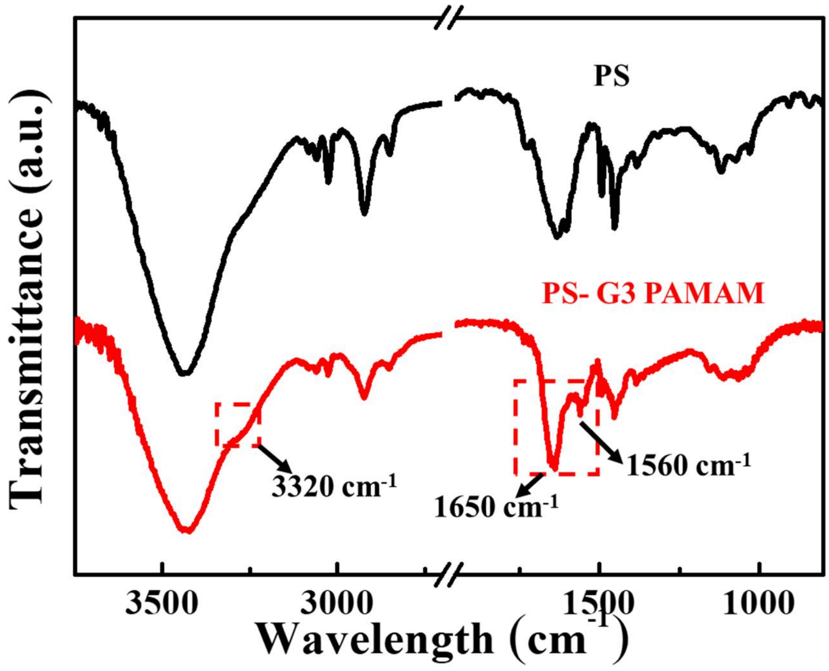

Figure S4. Infrared characterization of PS and PS-G3 PAMAM. The typical peaks at 3320 cm^{-1}, 1650 $cm^{-1}$and 1560 cm^{-1} are attributed to the N-H stretching vibrations of the unreacted terminal amino groups, C=O stretching (amide I) and N-H bending/C-N stretching (amide II) vibrations of G3 PAMAM dendrimers, respectively.

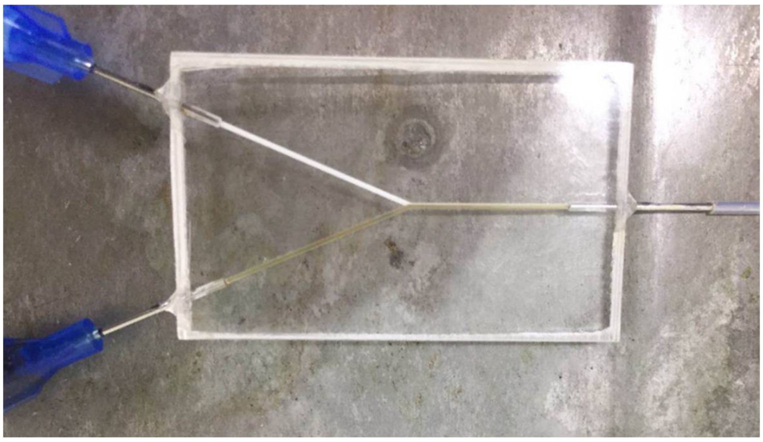

Figure S5. The microchip in microfluidic fabrication.

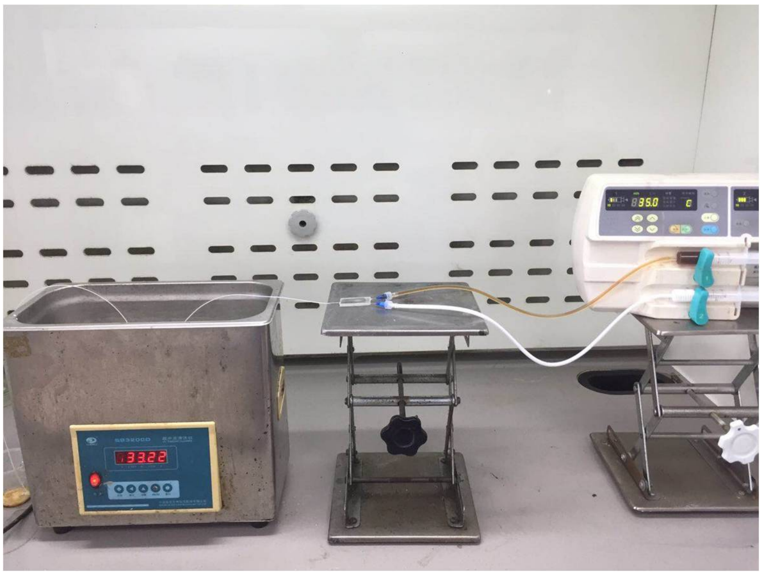

Figure S6. The microfluidic fabrication of fibers.

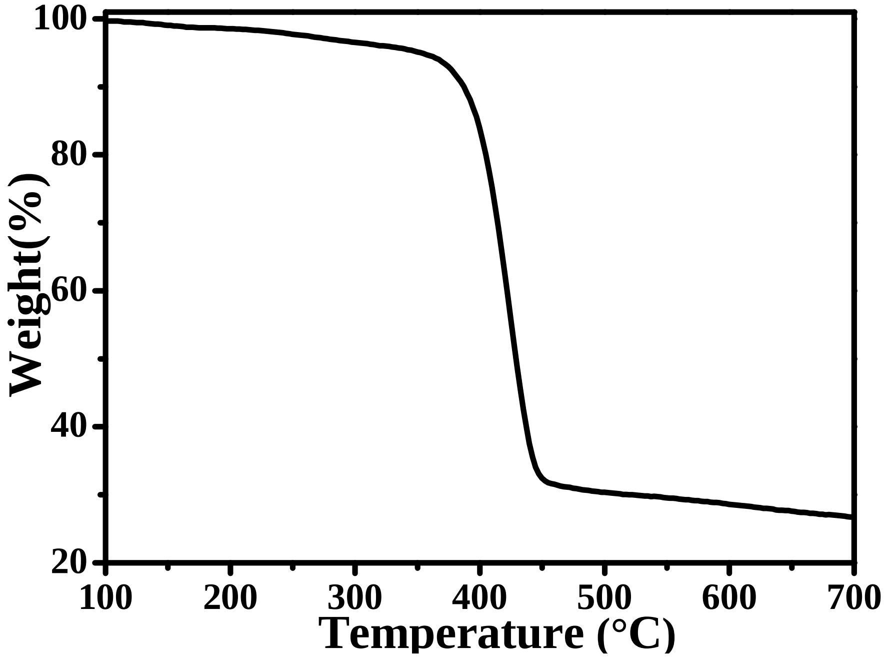

Figure S7. TGA curve of the GO/PS-G3 PAMAM hybrid fiber.

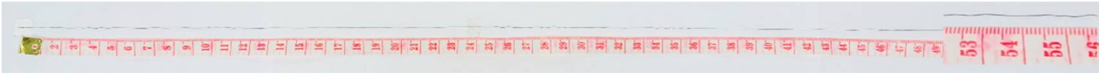

Figure S8. Photograph of VA-NiONSs/P-GF dry fiber with the length of 0.5 m.

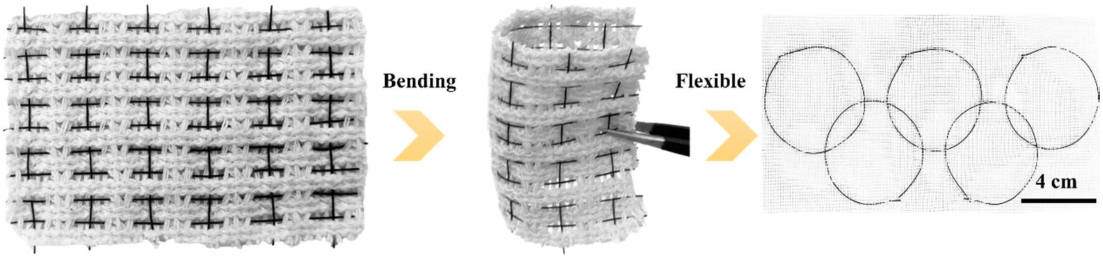

Figure S9. Fibers integrated into textiles with super flexibility.

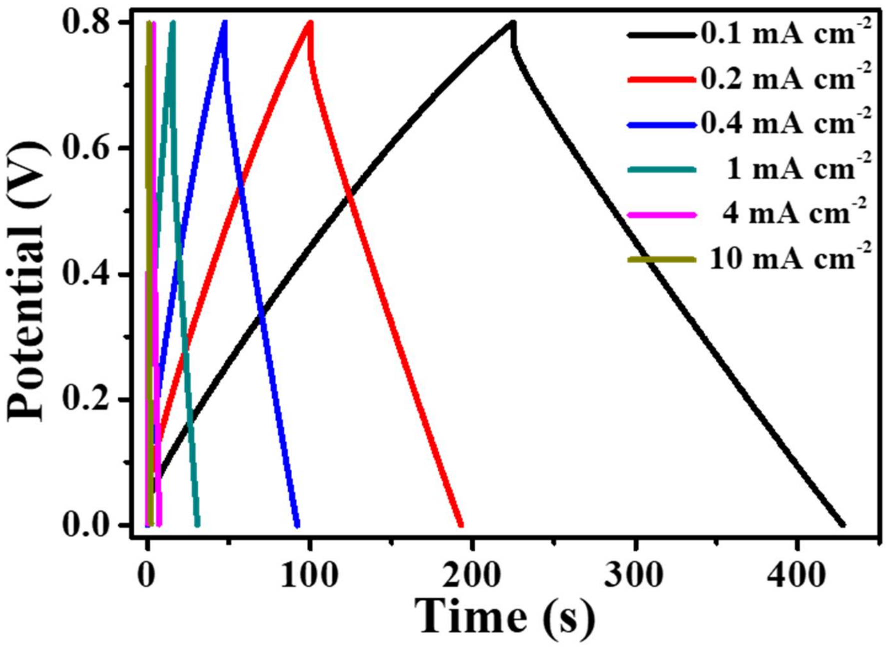

Figure S10. GCD curves of pristine graphene fiber-based micro-SC.

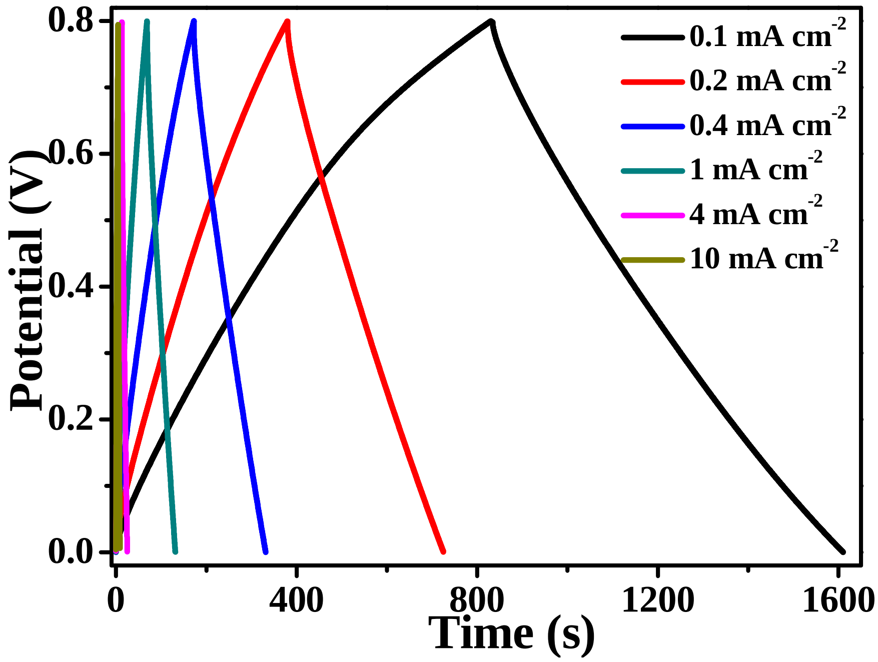

Figure S11. GCD curves of P-GF based micro-SC.

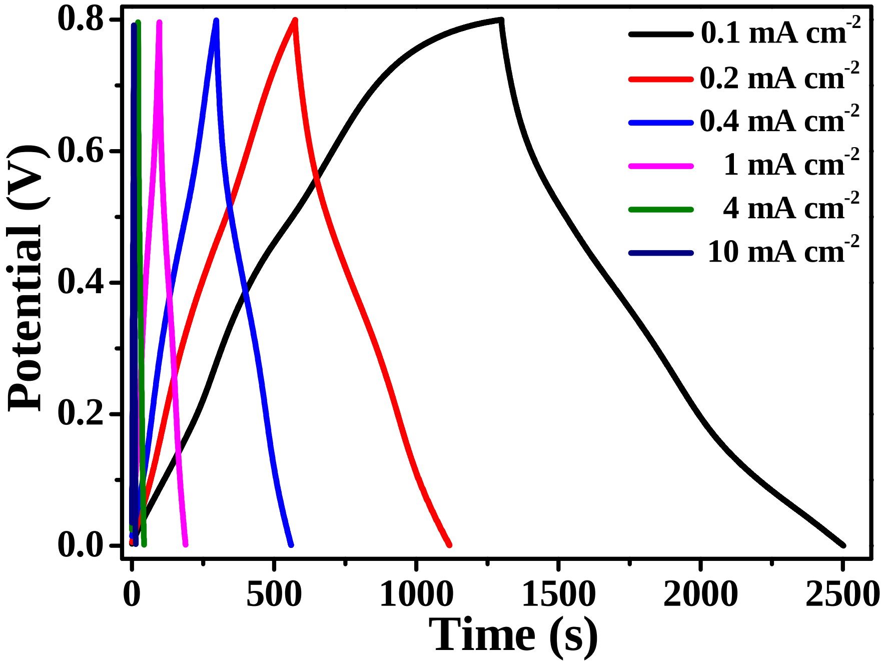

Figure S12. GCD curves of VA-NiONSs/P-GF based micro-SC.

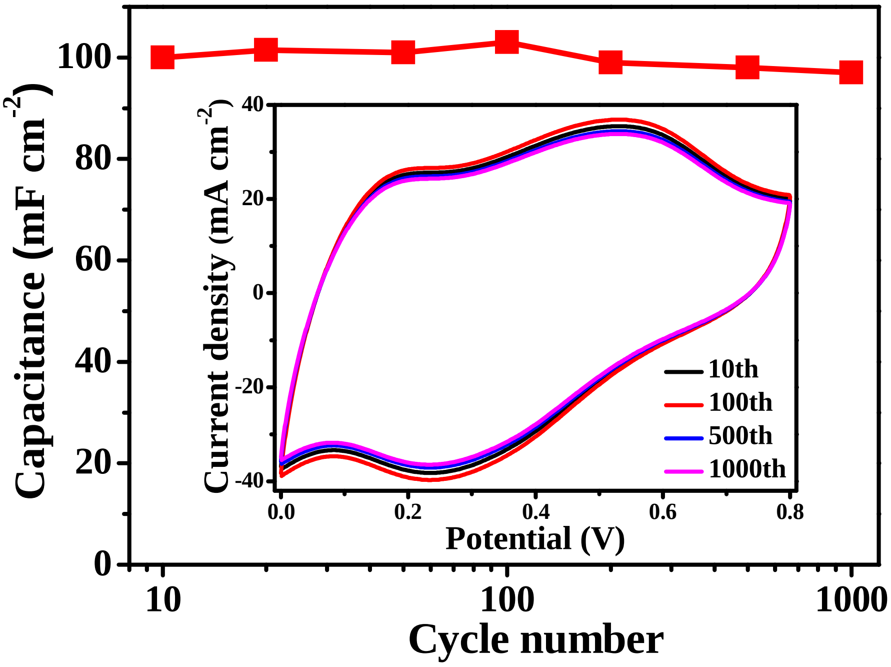

Figure S13. Capacitance retention of FMSCs under the bending angle of 180°.

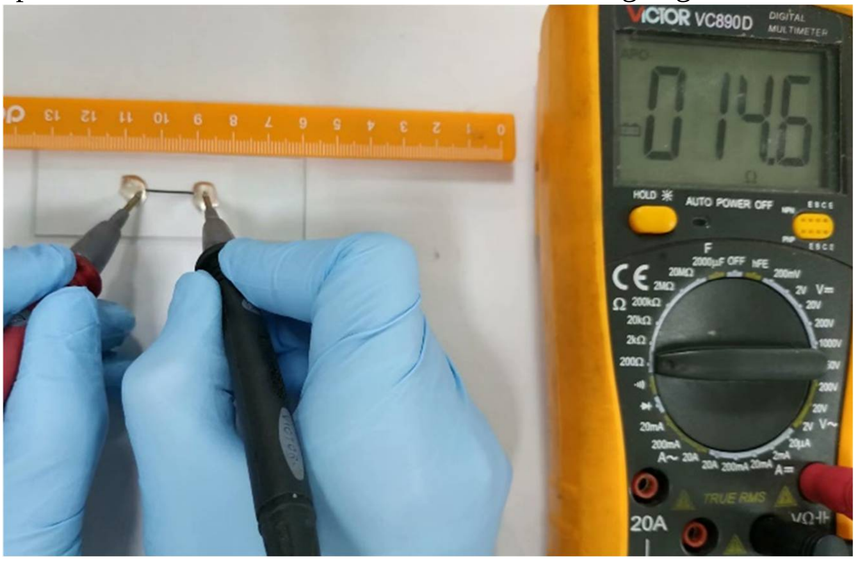

Figure S14. The electrical conductivity of VA-NiONSs-2/P-GF.

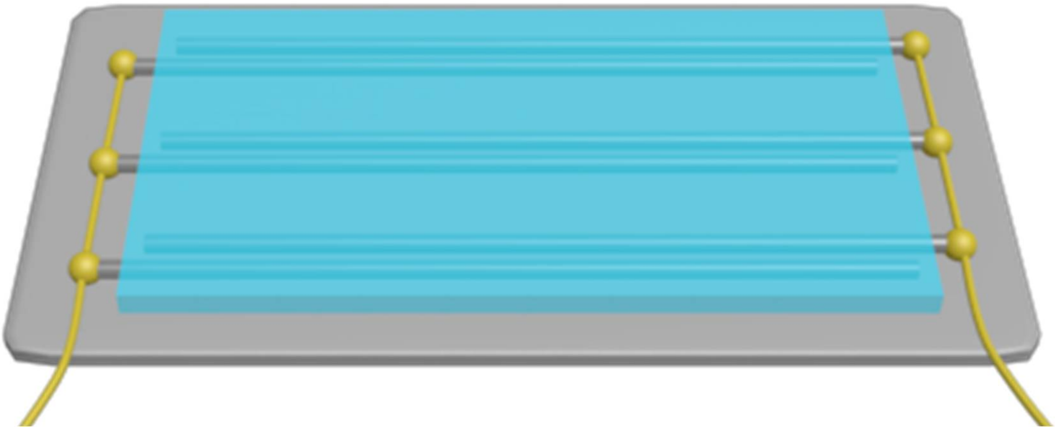

Figure S15. Schematic illustration of three FMSCs connected in parallel.

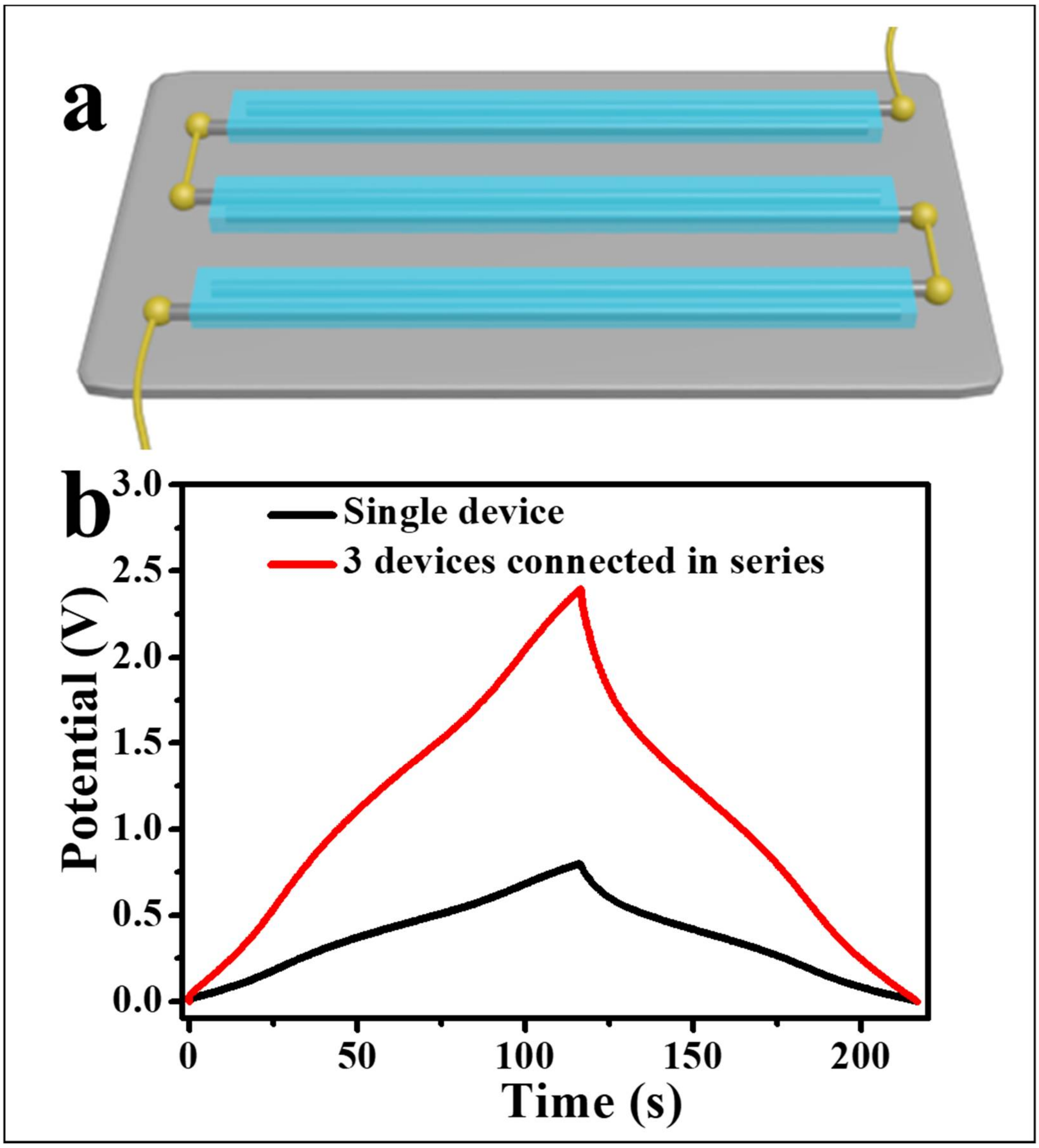

Figure S16. (a) Schematic illustration of three FMSCs connected in series. (b) GCD curves of single and three FMSCs connected in parallel.

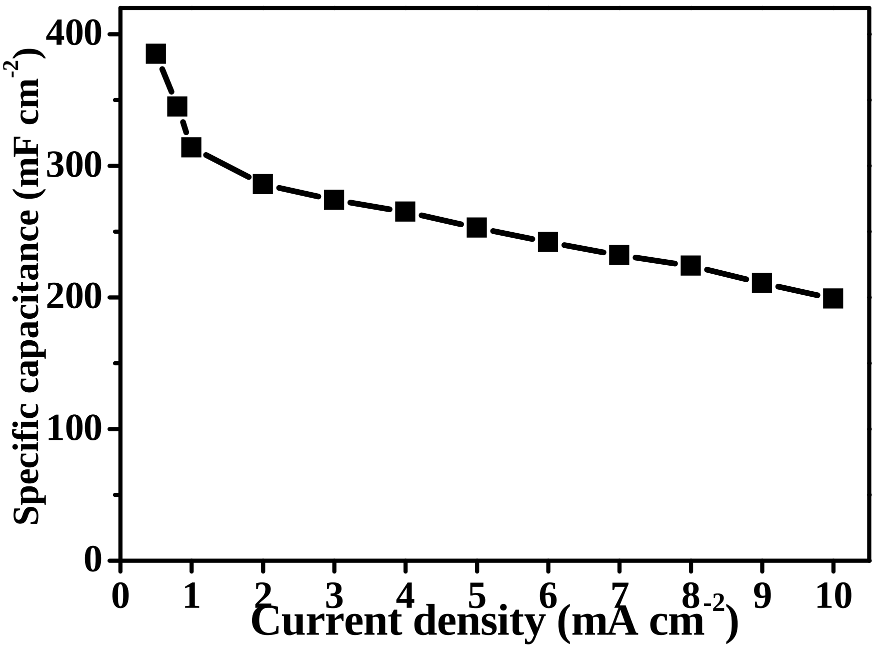

Figure S17. Calculated specific capacitances of VA-NiONSs/P-GF SC.

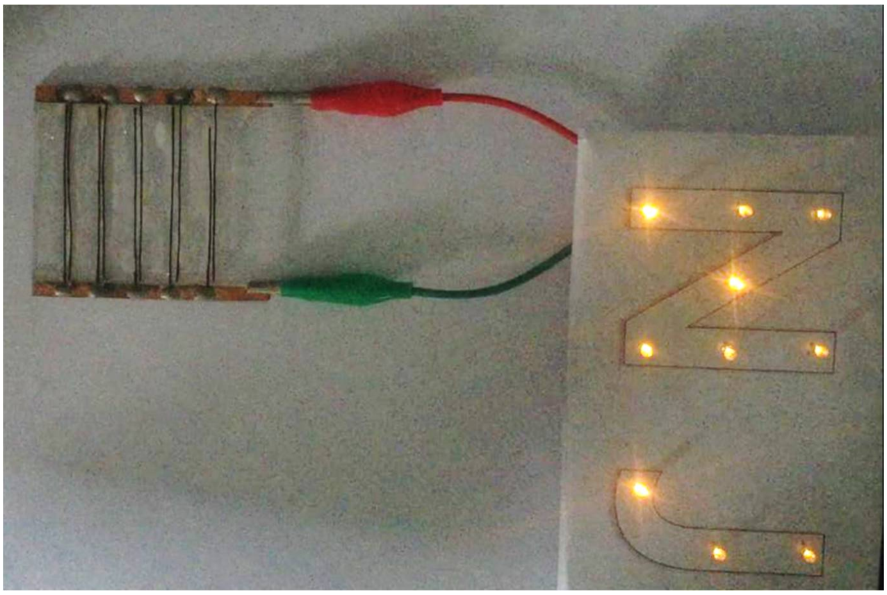

Figure S18. FMSCs integrated into flexible substrate to light up 10 LEDs.

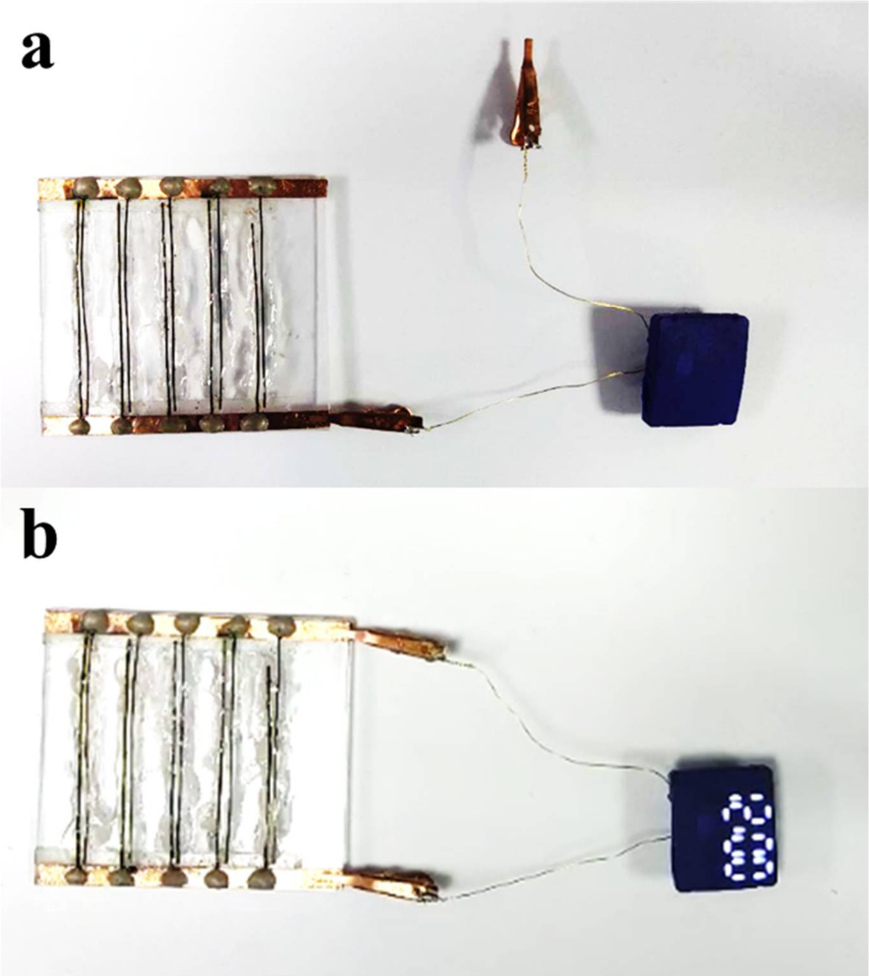

Figure S19. FMSCs integrated into flexible substrate to power watch.

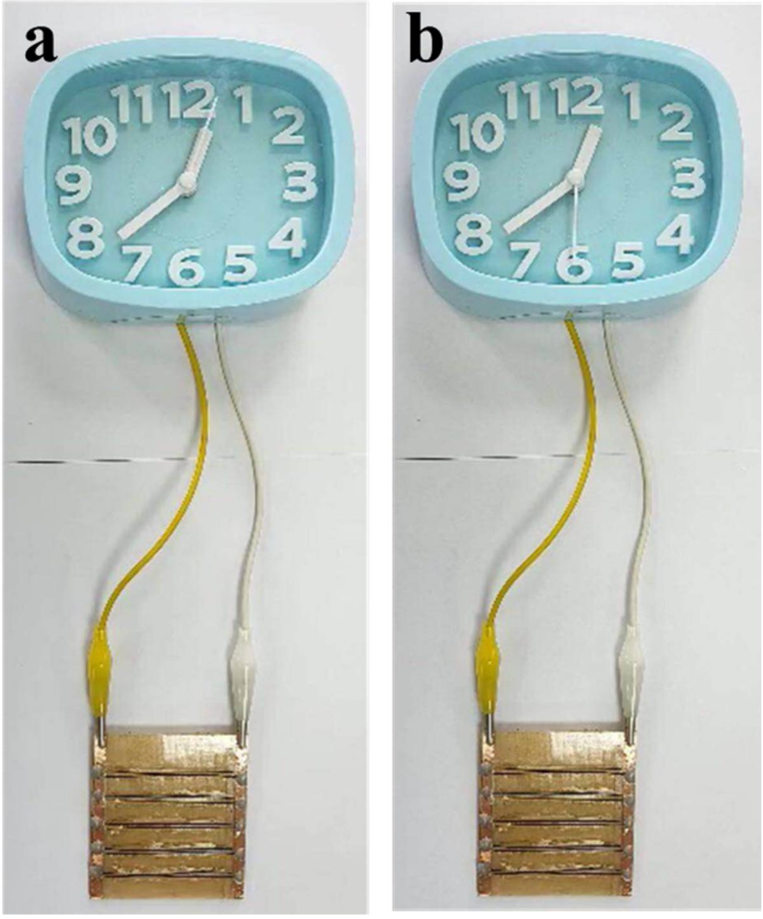

Figure S20. FMSCs assembled into flexible substrate to power clock.

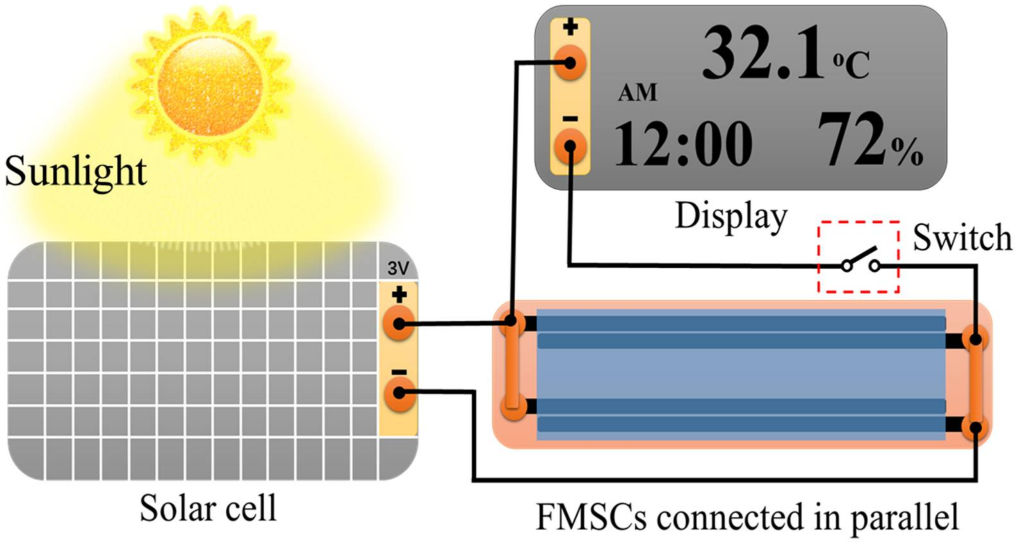

Figure S21. Schematic illustration of wearable self-powered system to light up display.

Table S1. EIS molding data. Parameter results simulated by the equivalent circuit in Figure 3d.

	R_0/Ω	C_1/mF $s^{n_1 -1}$	n_1	R_1/Ω	Z_w/Ω	C_2/F	n_2
GF	38.7	0.47	0.82	3.52	295	0.008	0.69
P-GF	28.9	0.45	0.73	3.99	69.3	0.035	0.87
VA-NiONSs /P-GF	32.5	0.38	0.75	4.67	47.2	0.053	0.93

Table S2. Parallel comparison with other works in terms of electrode materials, specific capacitance, energy density and electrolyte.

Electrode materials	Capacitance (mF cm^{-2})	Energy density (μWh cm^{-2})	Electrolyte	Reference
Graphene	1.7	0.17	PVA/H_2SO_4	9
CNTs/mesoporous carbon	39.7	1.77	PVA/H_3PO_4	18
CNT twisted fiber	92.1	/	PVA/LiCl	40
Graphene/Ni/Cu	133	78.1	PVA/KOH	10
CNT/ PEDOT:PSS	164.8	/	PVA/H_3PO_4	41
Graphene/CNTs	177	3.84	PVA/H_3PO_4	8
Graphene/PANI	119	37.2	EMIMTFSI/PVDF	23
Graphene/PEDOT:PSS	304.5	6.8	PVA/H_3PO_4	21
MnOx@TiN@CNTs	360	/	EMIMTFSI/PVDF	42
MXene	12	0.32	PVA/H_2SO_4	1
Graphene/Ni	72.1	1.6	PVA/H_3PO_4	45
Graphene/carbon dots	215	67.37	$EMIMBF_4$/PVDF	22
VA-NiONSs/P-GF	385<br>605.9	120.3<br>/	$EMIMBF_4$/PVDF<br>PVA/KOH	This work
